# Enhancing Structural Health Monitoring with Acoustic Emission Sensors: A Case Study on Composites under Cyclic Loading

**DOI:** 10.3390/s24020371

**Published:** 2024-01-08

**Authors:** Doyun Jung, Jeonghan Lee

**Affiliations:** Korea Atomic Energy Research Institute, 111 Daedeok-daero 989-gil, Yusenong-gu, Daejeon 34057, Republic of Korea

**Keywords:** acoustic emission testing, composites, *b*-value analysis, structural integrity assessment

## Abstract

This study conducts an in-depth analysis of the failure behavior of woven GFRP under cyclic loading, leveraging AE sensors for monitoring damage progression. Utilizing destructive testing and AE methods, we observed the GFRP’s response to varied stress conditions. Key findings include identifying distinct failure modes of GFRP and the effectiveness of AE sensors in detecting broadband frequency signals indicative of crack initiation and growth. Notably, the Felicity effect was observed in AE signal patterns, marking a significant characteristic of composite materials. This study introduces the I*b*_e_-value, based on statistical parameters, to effectively track crack development from inception to growth. The I*b*_e_-values potential for assessing structural integrity in composite materials is highlighted, with a particular focus on its variation with propagation distance and frequency-dependent attenuation. Our research reveals challenges in measuring different damage modes across frequency ranges and distances. The effectiveness of I*b*_e_-values, combined with the challenges of propagation distance, underscores the need for further investigation. Future research aims to refine assessment metrics and improve crack evaluation methods in composite materials, contributing to the field’s advancement.

## 1. Introduction

Structural health monitoring (SHM) has proven to be a valuable system for evaluating the abnormal conditions of structures such as nuclear power plants [1,2] and steam generators [3]. It primarily focuses on implementing damage identification strategies and is considered a promising system for assessing structural damage in real time [4,5]. SHM utilizes tools to assess changes in the structure, supporting engineers in maintaining the structural integrity of the facility continuously throughout its lifecycle. As a result, SHM systems have the potential to enhance reliability by reducing maintenance costs, preventing irreparable damage, and alleviating economic burdens. For these reasons, SHM is being applied in various fields such as wind power systems [6,7], high-pressure gas pipelines [8,9], and composite overwrapped pressure vessels (COPVs) [10]. However, despite the existence of various methodologies for sensor data analysis, they have not yet been widely used in the field. Therefore, continuous research is needed to refine these analysis techniques and evaluate their applicability and usefulness in real-world failure scenarios.

Most SHM systems are applied to assess abnormal conditions in locations that are inaccessible to non-destructive testing experts or during operation, making passive sensors suitable. Representative passive sensors include accelerometers and acoustic emission (AE) sensors [11,12,13,14]. Accelerometers are used to detect and measure the motion state of objects, including acceleration, velocity, and displacement. The impact of environmental factors is very important in the structural health assessment of engineering structures [15]. Particularly, factors such as temperature and noise can influence the dynamic characteristics of a structure and may obscure vibration signal changes caused by actual structural damage. This poses a particularly challenging problem for structural health assessment methods using accelerometers. AE sensors are used to assess physical changes, such as when AE manifests in the form of elastic waves, which occur when waves generated by stress changes inside the material reach the surface of the structure. AE signals that include information on abnormal conditions caused by environmental factors are important for the early detection and prevention of potential damage or defects within a structure [16]. This is because AE signals originate from the damage process itself. Therefore, by using AE parameters, it is possible to distinguish and analyze various issues within a structure, such as crack growth [17,18], corrosion [19], friction [20] and noise sources [16]. As a result, AE parameters become essential tools for maintaining the integrity of a structure and managing potential risks. For these reasons, AE sensors are applied to structural health monitoring (SHM) in various applications [16], including monitoring rock [16], concrete [21,22,23], and composite materials [24,25].

AE signals are converted into various AE parameters. The threshold-based AE-hit detection method functions by comparing the AE signal with a designated threshold, and any signal exceeding this threshold is identified as an AE-hit. Once an AE-hit is determined, three parameters become important: Hit definition time (HDT), hit lockout time (HLT), and peak definition time (PDT), which help in identifying the maximum amplitude and energy. Research is ongoing to interpret meaning from AE parameters. Ridge et al. [26] and Nair et al. [27] used amplitude and strength to assess damage in concrete samples, while Salamone et al. [28] applied statistical pattern recognition techniques to AE signals to identify the onset of damage. Ohtsu et al. [29] utilized AE energy and the Kaiser effect to study the damage state of RC beams under incremental cyclic loading. Colombo et al. [30] analyzed the fracture process in RC beams using the *b*-value based on the Gutenberg-Richter empirical formula [31], stating that changes in the *b*-value are associated with microcracks and macrocracks, and the *b*-value decreases as cracks grow. The Gallego research group applied AE testing to concrete [32,33] and FRP-reinforced wood beams [34] and proposed an SHM method based on the relationship between accumulated AE energy and plastic strain energy. This group empirically validated this methodology through several earthquake tests conducted on concrete structures, including RC structures with hysteresis dampers [35].

**Figure 1:**
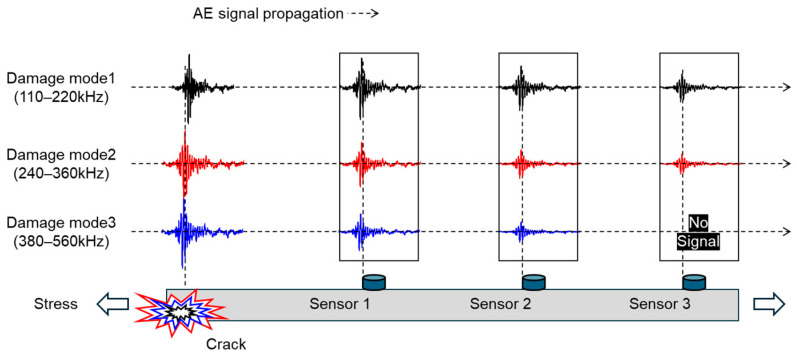
Effect of AE signal propagation characteristics from diverse damage modes on AE analysis: frequency dependence of attenuation.

Recently, the energy *b*-value (*b*_e_-value), which evaluates concrete cracks using AE hits and energy, has been successfully introduced [36]. However, its application in other areas, such as composite materials, has not yet been reported. Therefore, we assessed the applicability of the *b*_e_-value using woven GFRP (glass fiber reinforced polymer), commonly used in high-pressure piping and COPVs. Through this research, we aim to propose a method for supporting nuclear power systems or evaluating the structural integrity of mechanical systems that are planned to be supported. Analyzing bursts of AE signals generated from cracks through frequency analysis allows for the distinction of damage modes. Representative failure modes such as matrix cracking, interfacial failure, and fiber fracture are known to primarily occur in distinct frequency ranges of 100–220 kHz, 240–360 kHz, and 380–500 kHz, respectively [37,38,39,40,41]. Additionally, the frequency of AE signals exhibits characteristics of attenuation with respect to propagation distance, referred to as the frequency-dependent attenuation of AE signals (Figure 1) [42,43,44]. As a result, the ratio of amplitude, energy, and hits of AE signals measured from AE sensors installed at different locations from the crack changes. In other words, the *b*_e_-value may vary depending on the signal’s propagation distance. This phenomenon can occur not only in FRP but also in structures made using welding or cladding techniques.

However, there are still no research results that have been applied and evaluated in engineering structures or composite materials. In this study, we conducted destructive tests and AE testing using woven GFRP simultaneously. We varied the distance from the center hole to the sensors and observed changes in the *b*_e_-value. Through this research, we aim to evaluate the applicability of the *b*_e_-value. These results can be valuable not only for composite structures but also for mechanical structures where various abnormal conditions occur simultaneously. They can also provide important insights for improving the *b*_e_-value.

## 2. Experimental Procedure

### 2.1. Fracture Testing

We used woven GFRP to generate broadband AE signals from a single crack, which originated from various defects. The 2 mm thick woven GFRP, made of woven glass fibers and epoxy resin, was purchased from Murakami Dengyo Co., Ltd. in Shizuoka, Japan. This flat plate was produced using pre-preg woven glass fibers (52.3 vol%) and was laminated with 16 plies, then compression-molded at 170 degrees Celsius.

**Figure 2:**
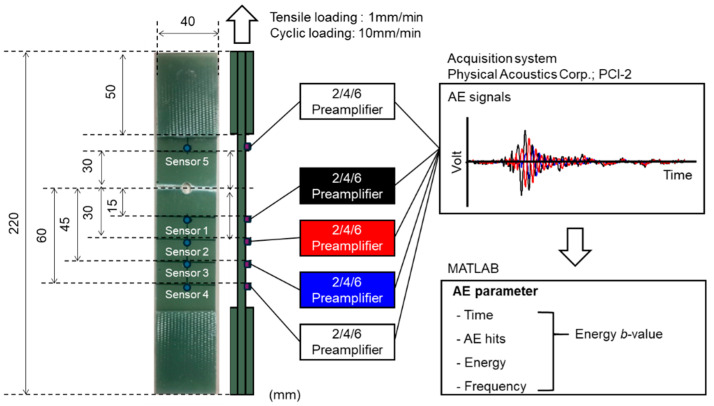
Experimental procedure for analyzing broadband AE signals in woven GFRP specimens with central hole.

Here is a step-by-step description of the experimental procedure (Figure 2):

- Material: GFRP sheets with a 2 mm thickness, comprising glass fibers and epoxy resin arranged in a plain-woven fabric configuration.
- Specimen Design: Woven GFRP plate specimens designed according to ASTM D3039 standards [45], including a center hole. The hole is created using a drill specifically designed for composite materials, resulting in a 2 mm-diameter hole at the center.
- Prevention of Damage: GFRP tabs are attached to each end of the specimen to prevent damage from the test jig.
- Tensile Testing: A universal testing machine (AGI; Shimadzu Corp., Kyoto, Japan) is employed for conducting the tensile tests. The crosshead speed is set at 1 mm/min (tensile loading), with cyclic loading at 10 mm/min.

**Figure 3:**
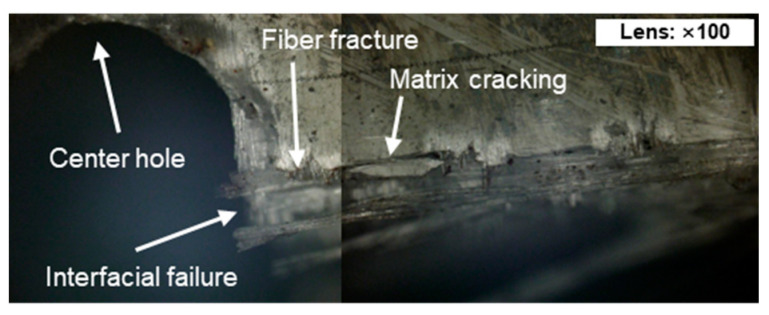
Fracture surface analysis of woven GFRP specimen under cyclic loading: observations of matrix cracking, interfacial failure, and fiber fracture.

Figure 3 shows the fracture surface of a woven GFRP specimen that failed due to cyclic loading. Near the center hole, matrix cracking, interfacial failure, and fiber fracture were observed. Consequently, the AE sensors detected signals of broadband frequencies originating from more than three defects [37,38].

### 2.2. AE Testing

AE testing was conducted using the following equipment and procedures:

- Digitizer and Preamplifiers: AE signals were recorded using a digitizer (Physical Acoustics Corp.; PCI-2, Princeton Junction, NJ, USA) with a per-channel sampling rate of 10 MHz during each test. The tests utilized 2/4/6 preamplifiers (Physical Acoustics Corp., Princeton Junction, NJ, USA) with a 40 dB_AE_ gain.
- AE sensor: For AE testing, a PICO sensor from the Physical Acoustics Corporation (PAC; Princeton Junction, NJ, USA) was selected. This sensor operated within the frequency range of 200 to 750 kHz. Three AE sensors, referred to as data sensors, were strategically positioned at various distances from the hole on each specimen (refer to Figure 2). Guard sensors (labeled as sensor 4 and sensor 5) were applied at both ends of the specimen to enhance signal reliability. The utilization of guard sensors in testing represented a strategic approach to enhance signal reliability. These guard sensors were deployed to identify sources originating outside the area of interest. The guard technique entailed placing data sensors within the area of interest, encircled by multiple guard sensors [16]. Such a configuration allowed for a clear distinction between waves emanating from the area of interest and those from external sources. AE waves from the area of interest reached the data sensors before impacting any of the guard sensors. In contrast, waves originating from outside struck at least one of the guard sensors before reaching the data sensors. This setup facilitated the exclusion of external noise, enabling a focused analysis of pertinent acoustic emissions from the specimen under test.
- Sensor attachment: The AE sensors were affixed to each specimen using silicon grease (HIVAC-G, Tokyo, Japan) and secured with vinyl tape.
- Fracture occurrence: Specimens experienced fractures near the center hole due to stress concentration, leading to Table 1, which summarizes the test conditions based on previous research for AE monitoring.
- Recording Criteria: AE signals exceeding 40 dB_AE_, as measured by the sensors, were recorded. This criterion helped filter out background noise and focus on relevant signals associated with the specimen’s behavior.

*2.3. b-Value Analysis*

The *b*-value analysis, initially developed by Gutenberg for seismological applications, is expressed by the formula [31]:

(1) $$\log_{10}\left(N\right)=a-b\times A$$

Here, *A* represents the amplitude of the AE signal in decibels (dB_AE_), *N* represents the total number of AE hits, *a* is an empirical constant, and *b* is the slope of the linear relationship. This analysis uses amplitude as a direct indicator of damage, associating a decrease in the *b*-value with crack growth. To further enhance the analysis, the I*b*-value [46] was introduced, considering specific statistical parameters of the amplitude distribution. The I*b*-value is calculated using the mean (*μ*), standard deviation (*σ*), lower amplitude (*μ* − *a*_1_ × *σ*), and upper amplitude (*μ* + *a*_2_ × *σ*) values:

(2) $$Ib=\frac{\log_{10}N\left(\mu -a_{1}\cdot \sigma \right)-\log_{10}N\left(\mu +a_{2}\cdot \sigma \right)}{\left(a_{1}+a_{2}\right)\cdot \sigma }$$

Sagasta et al. [36] proposed the energy *b*-value (*b_e_*):

(3) $$\log_{10}N({AE}_{e})=a-b_{e}\times \log_{10}AE_{e}$$

This proposed *b_e_*-value assesses local damage in reinforced concrete structures under dynamic loads. It is based on the traditional *b*-value but uses the true energy of AE signals instead of the peak amplitude. Experimental applications demonstrate its potential for damage assessment, with the *b_e_*-value responding when severe damage occurs in critical areas of the structure.

**Figure 4:**
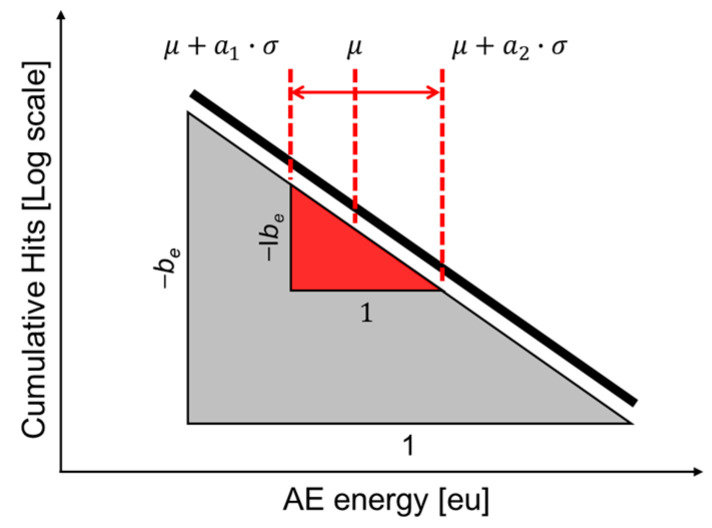
Representation of I*b*_e_-value for composite material in the study.

In the AE field, often considered microseismology, the *b*-value is extensively used to assess fracture processes. Similar to its application in engineering, the *b*-value in AE is associated with the number and amplitude of events. High *b*-values are linked to numerous small-amplitude AE events, like those from microcrack formation and slow crack growth, while low *b*-values indicate faster-growing or unstable crack formation. The *b*-value systematically changes during various stages of the fracture process, serving as a valuable indicator for estimating failure development. The composite material used in this study has a wide-frequency range, so it is necessary to select representative intervals and use their values. Therefore, Equation (2) was adopted into Equation (3) and defined as follows, where *a*_1_ = *a*_2_ = 3. The definition is illustrated in Figure 4.

## 3. Results and Discussion

**Figure 5:**
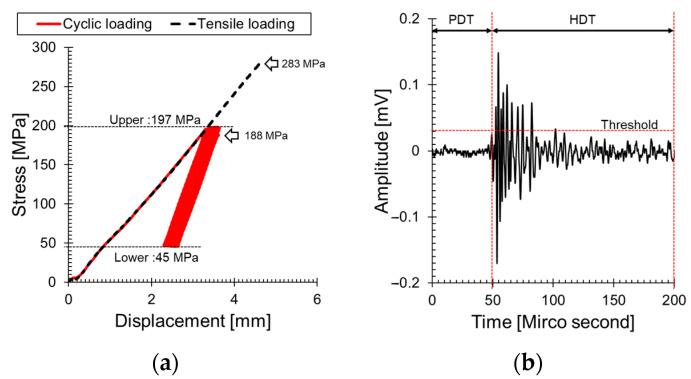
Behavioral analysis of woven GFRP under tensile and cyclic Loading: (**a**) stress–strain relationship and (**b**) acoustic emission signal response (See Table 1).

Figure 5a illustrates the stress–strain curve of GFRP under tensile loading and cyclic loading. A tensile strength of 238 MPa was calculated for GFRP under tensile loading. By setting 70% of this value as the upper limit (197 MPa) and 45 MPa, the initial occurrence of AE signals, as the lower limit, the cyclic loading range was determined. The GFRP failed during the cyclic loading test at the 423rd cycle, with a stress of 188 MPa. This stress is lower compared to tensile loading, as fatigue failure occurs due to repetitive loading, leading to crack initiation and propagation. Simultaneously, this experiment provides insights into how the *b*_e_-value responds to the occurrence and propagation of cracks.

During cyclic loading, AE signals are emitted from the crack growth to three sensors spaced at regular intervals from the center hole. The signals were recorded when each AE sensor exceeded 40 dB_AE_ (threshold-based AE-hits technique, Figure 5b). PDT and HDT values of the AE signal were recorded as 50 microseconds and 150 microseconds, respectively. After calculating the burst duration time of the recorded AE signal, the energy of that interval was computed.

**Figure 6:**
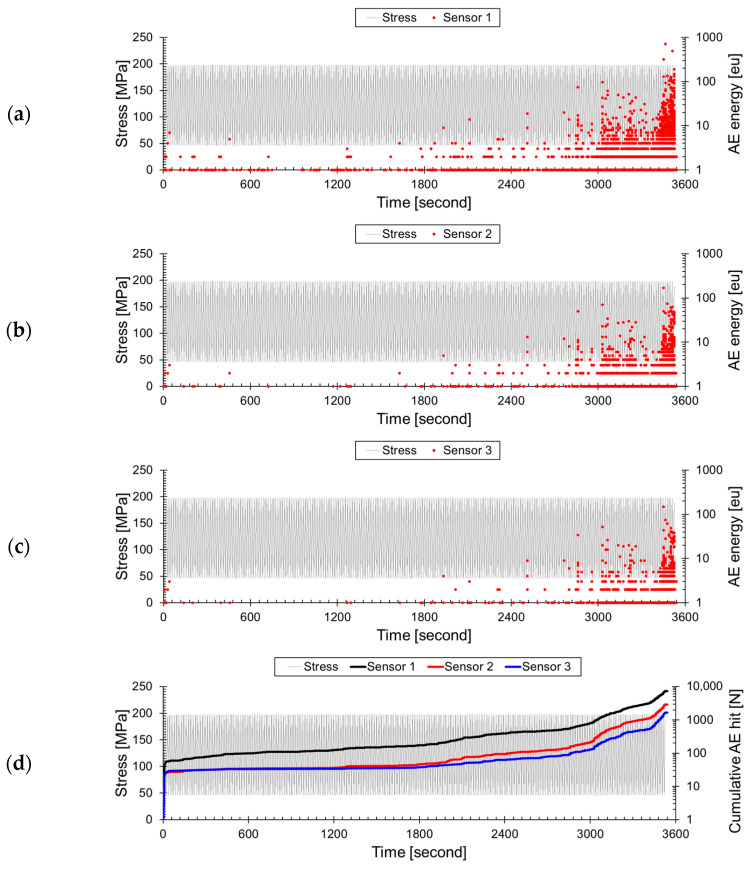
Variation in AE Energy with Increasing AE signal propagation distance during cyclic loading of GFRP: (**a**) 15 mm, (**b**) 30 mm, (**c**) 45 mm, and (**d**) cumulative hits at each propagation distance.

Figure 6 shows the results of converting the measured AE signals into AE energy during cyclic loading. In Figure 6a, AE energy progressively increased from 0 to 423 cycles. The AE energy of 7 eu recorded between 0 and 42 s is related to the formation of cracks. From 1931 s (9 eu) onward, a significant amount of AE energy is exclusively measured. This phenomenon is related to the Felicity effect, where AE signals are emitted before the previous maximum load. This occurrence is frequently observed in composite materials, including the GFRP specimen used in the test, indicating the growth of cracks. Until 3551 s, the maximum AE energy steadily increased to 707 eu, and this trend closely resembles the peak amplitude. Figure 6b,c shows the attenuated AE energy measured at propagation distances of 30 mm and 45 mm. These signals also consistently increased from the initiation of crack formation to the final failure. To clarify the attenuation effect, the cumulative hits of the calculated AE signals in Figure 6a–c were represented in Figure 6d. As the distance from the crack increased by 15 mm, the AE signals with AE energy decreased. However, the attenuation rate of the AE signals over time varied for each sensor. The failure behavior of composite materials is typically investigated to observe interface failure and fiber fracture occurring before matrix cracking [47,48]. Additionally, although the frequency ranges may vary among researchers [24,25,41], the general order is an increase in frequency corresponding to matrix cracking, interfacial failure, and fiber fracture. The AE signals generated near the final failure originate from matrix cracking, resulting in relatively low attenuation. On the other hand, a high attenuation ratio of matrix cracking and interfacial failure is observed in the crack initiation zone. For this reason, the difference in time–attenuation ratio (distance between the lines) is observed in Figure 6d.

**Figure 7:**
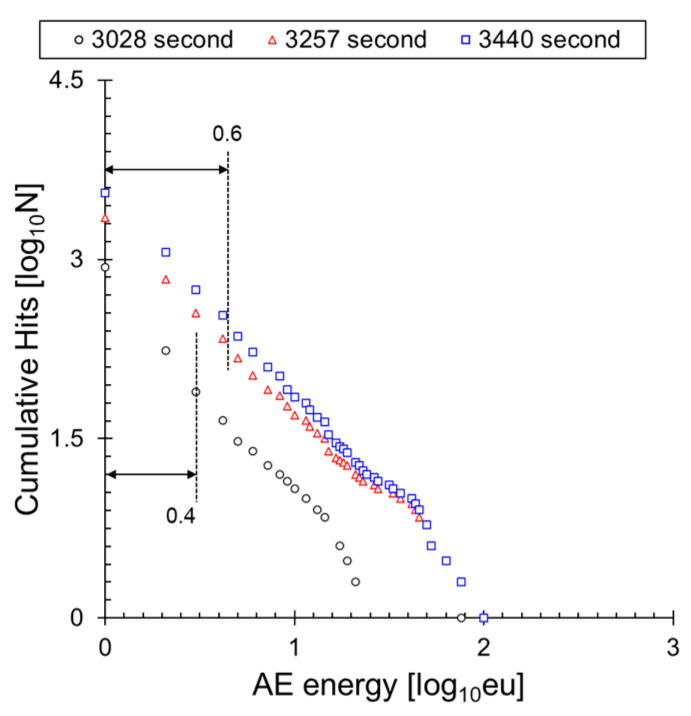
Change of frequency distribution.

Figure 7 shows the frequency distribution of the AE energy measured at Sensor 1 according to Equation (3). The *b*_e_-value proposed by Sagasta et al. [36] utilizes the slope of the entire frequency distribution. Cracks in various materials, including composite materials, typically occur after the initiation of microcracks, followed by the development of macrocracks. From the perspective of AE signals, microcracking refers to the phenomenon characterized by the occurrence of numerous small-sized cracks. Therefore, in regions where microcracking predominates, a significant number of AE signals with low energy are generated, leading to a decrease in the *b*_e_-value (3028 s to 3257 s). On the contrary, the occurrence of macrocracking, characterized by the formation of a single large-sized crack, results in an increase in the *b*_e_-value (3257 s to 3440 s). The trend of increasing or decreasing *b*_e_-values can be utilized to assess the structural integrity of a system, with an increase associated with microcracking and a decrease indicative of macrocracking. A similar occurrence is observed in the *b*_e_-value proposed by Gutenberg et al. [31]. When computing the I*b*_e_-value with Formula 3, we employed an interval of 0.02 for the frequency distribution and measured the slope after excluding non-accumulative intervals. We marked the representative interval of the frequency distribution, selected based on the mean and standard deviation, with an arrow at position 6. The slope was calculated using the method of least squares each time the AE signals reached multiples of 5.

**Figure 8:**
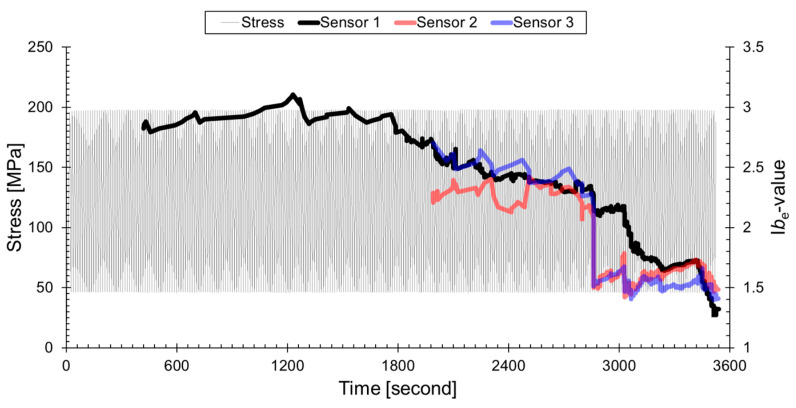
Time series analysis of I*b*_e_-values in relation to stress in woven GFRP: demonstrating the static evaluation approach for structural integrity.

**Figure 9:**
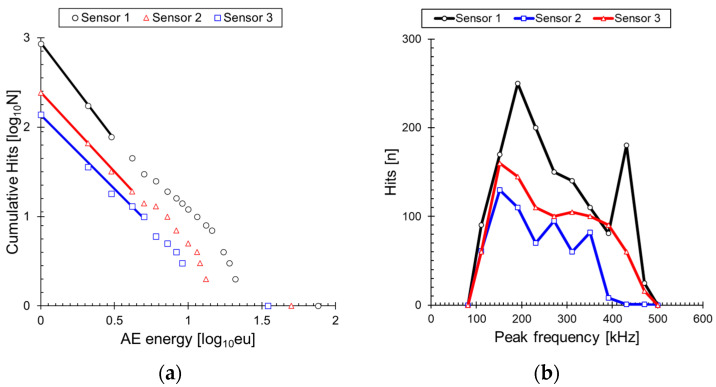
Frequency and attenuation characteristics of AE signals in woven GFRP: (**a**) AE energy frequency distribution and (**b**) peak frequency variation with propagation distance.

Figure 8 displays the time series plot of I*b*_e_-values along with stress. The calculation of I*b*_e_-values commenced after the accumulation of 200 data points. The I*b*_e_-values steadily decreased to 1.25 after the commencement of calculations. Unlike the dynamic approach of evaluating structural integrity using the trend of values, as seen in the *b*-value, the I*b*_e_-value employs a more static method that heavily relies on the actual values. In a normal condition, the I*b*_e_-value ranges from 3 to 2.75. As the material progresses from crack growth to final fracture, the I*b*_e_-value decreases from 2.75 to 1.25. Many researchers assess the structural integrity by designating a specific value of I*b* as the threshold for discontinuing the use of the structure. However, this study does not provide evaluation metrics as the I*b*_e_-value was calculated using only a limited number of GFRP specimens. The most significant finding in this study is our confirmation of the powerful performance of the I*b*_e_-value, demonstrating its potential for evaluating the structural integrity of composite materials using this method. On the other hand, the I*b*_e_-value exhibited an overall decreasing trend as the propagation distance increased up to 45 mm. This phenomenon is attributed to the frequency dependence of the damping in the previously mentioned composite material. For a more detailed explanation, the AE energy frequency distribution measured up to 3028 s is illustrated in Figure 9a, concurrently with the representation of representative intervals based on Formula (3). Figure 9b illustrates the variations in peak frequency with propagation distance under identical conditions. The signals measured at Sensor 1 exhibited uneven attenuation over a propagation distance of 30 mm, leading to a change in the curve’s shape and consequently causing a variation in the I*b*_e_-value. This phenomenon arises in the cracks of GFRP that exhibit broadband signals due to factors such as matrix cracking, interfacial failure, and fiber fracture. Each damage mode is characterized by a unique frequency and exhibits varying rates of attenuation. Consequently, it is observed that signals in the high-frequency range attenuate more rapidly than those in the low-frequency range [42,43,44]. This propagation characteristic is clearly depicted and validated in Figure 9b. Thus far, we have utilized the I*b*_e_-value results obtained from AE signals emitted from the center hole as a basis for discussing the applicability of composite materials. It has been confirmed that the I*b*_e_-value, along with previously proposed metrics such as *b*_e_-value and I*b*_e_-value, can be used to evaluate cracks in composite materials. However, since the values vary with the propagation distance, more extensive research is needed to understand this phenomenon.

## 4. Conclusions

In this study, we have conducted a comprehensive analysis of the failure behavior of woven GFRP under cyclic loading, utilizing AE sensors to monitor the progression of damage.

(1) Our findings demonstrate that woven GFRP exhibits distinct failure modes when subjected to cyclic loading. The effectiveness of AE sensors in detecting broadband frequency signals from various failure modes provided valuable insights into the initiation and growth processes of cracks. A notable observation was the distinct presence of the Felicity effect, a characteristic of composite materials, in the AE signal patterns. Additionally, we observed that the AE signals attenuated with increasing distance from the crack source, which in this case was the center hole.
(2) Employing the I*b*_e_-value, which relies on statistical parameters, we were able to adeptly index the evolution of cracks from their formation to growth, originating from the center hole. One of the significant discoveries of this study is the potential application of the energy *b*-value in assessing the structural integrity of composite materials. However, we noted that the I*b*_e_-value varied with the increase in propagation distance, a phenomenon attributed to the frequency dependence of attenuation.

This research has brought to light potential challenges that may be encountered when measuring different damage modes across various frequency ranges and at different propagation distances. The demonstrated effectiveness of I*b*_e_-values, coupled with the identified challenges related to propagation distance, emphasizes the need for further investigations. These future studies would aim to refine assessment metrics and enhance the reliability of crack evaluation methods in composite materials.

## Figures and Tables

**Table 1:** Test conditions for AE testing.

Threshold		Amplifier		Analog Filter		Sampling Condition	
Type	dB_AE_	dB_AE_	Lower	Upper	Rate	PDT	HDT *
Fixed	35	40	1 kHz	1 MHz	10 MHz	50 μsec	1.5 k μsec

* HDT: recording time for each acoustic emission (AE) signal after the AE signal exceeded the threshold value.

## Data Availability

Data are contained within the article.
